# Optimizing Transcutaneous Spinal Cord Stimulation: An Exploratory Study on the Role of Electrode Montages and Stimulation Intensity on Reflex Pathway Modulation

**DOI:** 10.3390/bioengineering12040410

**Published:** 2025-04-12

**Authors:** Shirin Madarshahian, Michael Trakhtorchuck, Tatiana Guerrero-David, Kristin Gustafson, James S. Harrop, Caio M. Matias, M. J. Mulcahey, Alessandro Napoli, Alexander Vaccaro, Mijail Serruya

**Affiliations:** 1 Raphael Center for Neurorestoration, Thomas Jefferson University Hospital, Philadelphia, PA 19107, USA; michael.trakhtorchuk@jefferson.edu (M.T.); alessandro.napoli@jefferson.edu (A.N.); 2 Center for Outcomes and Measurement, Thomas Jefferson University Hospital, Philadelphia, PA 19107, USA; tatiana.guerrero@jefferson.edu (T.G.-D.); maryjane.mulcahey-hershey@jefferson.edu (M.J.M.); 3 Physical Medicine and Rehabilitation, Thomas Jefferson University Hospital, Philadelphia, PA 19107, USA; kristin.gustafson@jefferson.edu; 4 Department of Neurosurgery, Thomas Jefferson University Hospital, Philadelphia, PA 19107, USA; james.harrop@jefferson.edu (J.S.H.); caio.matias@jefferson.edu (C.M.M.); 5 Department of Orthopedic Surgery, Rothman Orthopaedics, Philadelphia, PA 19107, USA; alex.vaccaro@rothmanortho.com

**Keywords:** transcutaneous spinal cord stimulation (tSCS), personalization, electrode placement montages, upper extremity, paired pulse, dorsal root reflex pathway, patient-centric

## Abstract

Transcutaneous spinal cord stimulation (tSCS) is a promising non-invasive method to improve motor function in individuals with spinal cord injury (SCI) by enhancing spinal reflex pathways. This study aimed to investigate the effects of different tSCS electrode placement montages and targeted spinal levels on neurophysiological responses such as spinally evoked motor responses (sEMRs), dorsal root reflex activation, and muscle recruitment in individuals with SCI and healthy controls to optimize stimulation strategies for motor recovery. Five participants (three individuals with SCI and two controls) underwent transcutaneous spinal cord stimulation using various electrode montages, target spinal level stimulation, and single- and paired-pulse paradigms. Electromyographic responses were analyzed to determine sEMR threshold, amplitudes, and paired-pulse attenuation. Different spinal levels and spatial configurations of electrode placements influenced the sEMR threshold and incidence of sEMR across all participants. Paired-pulse analysis showed more pronounced second-pulse attenuation in SCI participants (48 ± 36%) than in controls (12 ± 20%, *p* = 0.0425), with distinct trends observed across montages and muscle groups. These findings suggest that spinal level, electrode configuration, and paired-pulse effects are key factors in personalizing tSCS, informing the development of patient-centered therapeutic strategies. Future studies with larger and more diverse cohorts are needed to validate and expand these findings.

## 1. Introduction

Transcutaneous spinal cord stimulation (tSCS) is a non-invasive technique designed to enhance motor function in individuals living with spinal cord injury (SCI) by activating spinal reflex pathways through depolarization of afferent fibers [1,2]. With advancements in technology and the emergence of closed-loop tSCS systems [3,4], targeted approaches that can dynamically adjust stimulation parameters in a task-specific manner became a reality [3,4]. However, a deeper understanding of the physiological interactions between different parameters of SCS and spinal cord motor control at and below the level of injury is still needed to fully harness the potential of this intervention. This understanding can support design of stimulation programs that are tailored to individuals based on their unique physiological responses, thereby enabling a patient-centric approach that can enhance the therapeutic effects of this modality [4].

Despite the encouraging results in tSCS, many knowledge gaps still exist, such as the effects of changing electrode placement or stimulation parameters. Furthermore, differences in methodologies across studies limit the ability to draw definitive conclusions, even when targeting similar recovery outcomes [2,5]. For instance, multiple studies have focused on postural tasks such as standing [6,7,8], yet electrode placement differed among them in terms of specific levels and montages. Similarly, electrode placement differed across studies focused on the upper extremity, without a well-justified systematic approach. Even studies focused on similar recovery endpoints and patient populations differed in electrode placement: using single-site stimulation [9] or multi-site stimulation [10], or stimulating different spinal levels in the same patient population (i.e., those with similar AIS level of injury). Placement of the anode (the electrode defined as anode for the first phase of a biphasic pulse) also varied across studies, with common sites reported as the bilateral iliac crests [6,11], bilateral clavicles [12], anterior neck [13], bilateral abdomen [14], over thoracic level of spine [15] and midline on abdomen [16]. Once again, determination of electrode placement and montages was not based on systematic rationale.

Over the past decade, stimulation amplitude has been reportedly adjusted to sub-motor [3,17], motor [2], or supra motor [18] thresholds and each were reported to have specific functional and recovery outcomes. Amplitude was determined using different criteria, such as tolerance capacity [3,19], producing reflex attenuation [11], producing paresthesia by inducing sensory sensation below the motor threshold [17], a percentage of maximal response amplitude in musculature activity level, achieving a certain level of functional outcome [10], evoking a step-like movement [18], or based on the posterior root–muscle reflex amplitude [16]. However, no published study has objectively quantified the effect of different stimulation intensities on neurophysiological states in persons with SCI or in healthy individuals.

It is well understood that to optimize tSCS benefits, electrodes must be strategically placed to target key muscles and uniformly stimulate motor neuron pools [16]. Multiple studies have explored the effects of electrode placement on spinally evoked motor responses (sEMR). One study [20] examined how electrode positioning and montages influence the intensity required to elicit sEMR in lower limb muscles. Anode electrode placement over the anterior neck has been shown to elicit larger spinal reflex responses in upper limb muscles compared to bilateral placement over the iliac crest, with no reported differences in discomfort between these montages at similar intensities [11,21]. In one study [11], four different anode locations (anterior neck, bilateral iliac crest, bilateral clavicle, and 4 cm below the cathode on posterior neck) were explored. For the anterior neck location, the cathode was moved from C6 to C7 and from C7 to T1 and the optimal cathode location was chosen and held constant for each participant to allow further exploration of different anode locations. Out of four configurations, the study reported that placing the anode at T1 (4 cm below the cathode on the posterior neck) yielded the highest motor evoked potential (MEP) in hand musculature.

While individual patient assessment and clinical needs are the most common approaches taken to inform the application of Functional Electrical Stimulation (FES) and Epidural Stimulation (ES) programs, in a research environment, establishing the efficacy of an intervention such as tSCS requires standardized protocols that guide systematic decisions. Our goal is to establish a foundation for personalized tSCS, which will enable a more targeted and effective therapeutic approach. This study examined the immediate neurophysiological effects of tSCS, focusing on the effects of electrode placement montages and target spinal level on reflex responses, evoked motor response amplitudes, and muscle recruitment in the upper extremities. Specifically, we aimed to determine whether electrode montage and targeted spinal level can be optimized to achieve higher responsiveness across muscles and what differences result in terms of engagement of dorsal root fibers, while accounting for individual anatomical variations and differences between persons with and without SCI.

## 2. Materials and Methods

This study employed a quasi-experimental design. Five participants were enrolled in this study, comprising three individuals with SCI (complete or incomplete) and two individuals without spinal cord injury (non-SCI “controls”). The selection of participants adhered to specific inclusion and exclusion criteria, which are detailed in Table 1. Demographic information for the SCI participants is summarized in Table 2. The non-SCI control subjects consisted of a 27-year-old male and a 34-year-old female, for both of whom was confirmed the absence of any neurological impairments or relevant medical histories that could influence study results.

### 2.1. Initial Electrophysiological Assessment of Neural Connectivity

The participants with SCI participated in a Scanning Session designed to assess muscle excitability through the application of electrical stimulation to peripheral nerves. This procedure utilized a Chattanooga Continuum portable neuromuscular electrical stimulation (NMES) unit (Enovis™, Lewisville, TX, USA). The primary goal of the Scanning Session was to identify muscle responsiveness by administering transcutaneous electrical stimulation to areas of the body situated below the level of motor impairment. For participants to remain eligible for the study, it was required that at least one of their muscles exhibit a measurable response to the electrical stimulation.

Following the Scanning Session, participants who remained eligible proceeded to one or two subsequent study visits focused on determining the neurophysiological effects of tSCS associated with various montages and targeted spinal levels. During these visits, multiple factors relevant to SCS were systematically explored, including electrode placement (various montages and spinal levels) in single pulse and paired-pulse paradigms to assess the reflex pathways affected by the stimulation and effects of different levels of stimulation intensities on reflex responses and muscle recruitment.

### 2.2. Electrode and Stimulation Setup

Transcutaneous spinal cord stimulation (tSCS) was administered using self-adhesive hydrogel surface electrodes (PALS^®^, Axelgaard, Fallbrook, CA, USA), with stimulation delivered to the spinal column via the Reynolds Innovative Spinal Electrical Stimulation (RISES) system [3]. The RISES system is an advanced multi-sensor closed-loop tSCS platform developed at the Raphael Center for Neurorestoration, integrating several key hardware components: the Xcite2 Functional Electrical Stimulator (Restorative Therapies Inc., MD, USA), the Trigno Avanti Sensor system (Delsys, MA, USA) for electromyography (EMG), the MTw Awinda motion capture system (Movella, NV, USA), and a Windows laptop equipped with custom in-house developed RISES software (Version 1.4). The RISES system offers both open-loop and closed-loop stimulation modes. The closed-loop mode can adjust stimulation parameters dynamically based on real-time movement data, allowing for enhanced responsiveness during therapy. A distinct feature of this system is its ability to unify EMG and motion capture data for activity recognition, further facilitating task-specific movement analysis.

Single-pulse tSCS was delivered in the form of a charge-balanced, symmetric, biphasic rectangular pulse with a duration of 1 ms per phase (resulting in a total biphasic pulse width of 2 ms). In our experimental setup, the paravertebral electrode served as the cathode during the first phase and the anode during the second phase of the biphasic pulse. To maintain consistency, the paravertebral electrode was referred to as the “cathode” and the return electrode as the “anode”. For each stimulation intensity, three single pulses were delivered with an interstimulus interval of 3 s.

For investigations focused on reflex attenuation, pairs of stimuli with an interstimulus interval of 50 ms were applied at varying amplitudes for each participant. The amplitude range for stimulation was individualized and established according to each participant’s maximum comfortable tolerance level, which could differ based on the specific electrode montage and targeted spinal level.

Five distinct montages were employed to thoroughly investigate the differences between single-site and multi-site electrode arrangements, variations in anode electrode placements, different electrode sizes, and the anterior versus posterior positioning of electrodes on the body. A summary of the target spinal levels, electrode placement montages, and electrode sizes is provided in Table 3.

**Figure 1:**
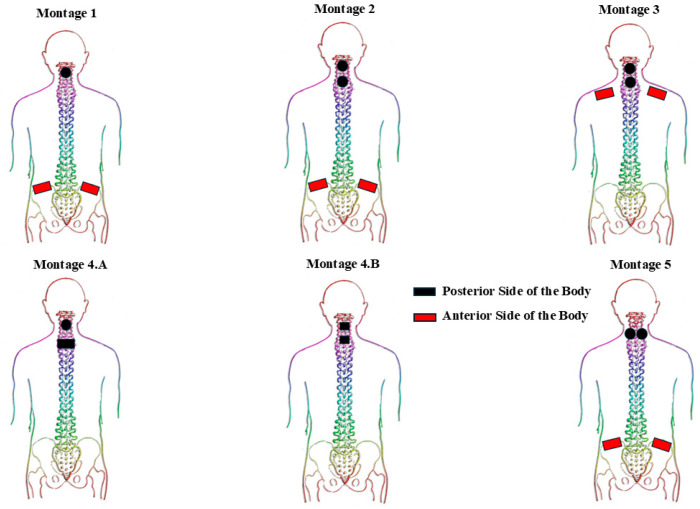
Illustrates various configurations for upper extremity tSCS.

Figure 1 provides a schematic representation of different montages with distinction of electrode placements anteriorly and posteriorly. Briefly, Montage 1 consisted of a circular electrode positioned at the spinal level of interest (based on the method elaborated in Section 2.4), accompanied by two rectangular interconnected electrodes placed bilaterally on the anterior iliac crest. Conversely, Montage 2 includes two interconnected circular electrodes at two spinal levels of interest (one electrode placed at each level), and a pair of interconnected rectangular electrodes positioned bilaterally on the anterior iliac crests. Montage 3 utilized two interconnected circular electrodes at two spinal levels of interest (one electrode at each level), and a pair of interconnected rectangular electrodes placed bilaterally on the anterior clavicles. Montage 4A involved a circular electrode placed at the spinal level of interest with a rectangular electrode positioned 4 cm inferior to the cathode electrode (both on the posterior side of the body). In contrast, Montage 4B employed two square electrodes located on two separate spinal levels of interest on the posterior side of the body. Lastly, Montage 5 consisted of two interconnected circular electrodes placed at the spinal level of interest, separated by an interelectrode horizontal distance of 3 cm, along with two interconnected rectangular electrodes applied bilaterally on the anterior iliac crests.

### 2.3. Data Collection Procedure

The electromyographic (EMG) activity elicited from sEMR was recorded using wireless Delsys EMG sensors (Delsys Trigno^®^, Road Natick, MA, USA) on the left and right Biceps Brachii (BB), Triceps Brachii (TB), Flexor Digitorum Superficialis (FDS), Extensor Digitorum Communis (EDC), and First Dorsal Interosseous (FDI) muscles. Each EMG sensor was positioned centrally over the muscle belly and oriented parallel to the long axis of each muscle to ensure optimal signal capture.

Prior to electrode placement, the skin was shaved and cleansed with alcohol to eliminate sweat, dead skin cells, and dirt that could interfere with the quality of the EMG signals. The analog signals produced by the EMG sensors were recorded, amplified, and subjected to filtering using a Butterworth filter. Specifically, a 2-pole high-pass filter with a corner frequency of 20 Hz and a 4-pole low-pass filter with a corner frequency of 450 Hz were employed to effectively eliminate noise and artifacts outside the frequency range of interest. After filtering, the filtered analog signals were digitized at 2048 samples per second at a resolution of 16 bits per sample which facilitates high-quality data acquisition necessary for detailed analysis. After initial data acquisition, all EMG data underwent an additional offline low-pass filtering step at 500 Hz, also utilizing a second-order Butterworth filter.

### 2.4. Experimental Protocol

We initiated our investigation by identifying differences in upper extremity muscle responses elicited by stimulation across various spinal levels in each participant. Utilizing Montage 1, levels tested encompassed the intervertebral regions between C3-4, C4-5, C5-6, C6-7, and C7-8. At each stimulation site, single-pulse stimulation was administered with gradually increasing intensities, advancing in increments of 5 mA. The stimulation intensity was increased until the participant’s maximum tolerance level (the point at which they reported it as too uncomfortable to tolerate). Subsequently, paired-pulse stimulation was delivered at each site, beginning with an intensity of 5 mA and progressing up to each participant’s maximum tolerance level. The absolute range of intensities used in this study was (5 mA–55 mA).

For single-biphasic-pulse trials, each stimulation intensity was delivered three times (repetitions), with an interval of approximately 3 s between each pulse. We determined the most responsive levels using specific criterion: a level was considered most responsive if it elicited sEMR activity in at least one muscle at lower intensities than other levels.

Additionally, we quantified the first stimulation intensity at which an sEMR emerged (“sEMR threshold intensities”) and the amplitude of muscle activation corresponding to these sEMR threshold intensities, the threshold amplitude values (“tAmpValue”).

Subsequently, for each participant we implemented both single-pulse and paired-pulse paradigms at the most responsive spinal level to investigate single-site montages. We also tested multi-site montages by stimulating the first and second most responsive levels simultaneously.

The effects of frequency, pulse-width and waveform type were explored for two SCI participants (SCI02 and SCI03), but not for SC101 or any control subjects. For pulse-width exploration, single biphasic pulses with different durations were delivered. For each pulse phase duration (250 µs, 500 µs, 1000 µs), stimulation was increased in increments of 5 mA, starting from 5 mA to maximum tolerance level. For each stimulation intensity, three biphasic pulses were delivered.

### 2.5. Data Analysis

#### 2.5.1. Cumulative Incidence of sEMR Across Levels and Montages

For each stimulation intensity, a data window spanning from −50 ms to 100 ms relative to the pulse onset was selected for analysis. From these data, pulse-triggered averages for three trials corresponding to each specific stimulation intensity were calculated. Subsequently, the EMG signals underwent preprocessing using a fourth-order Butterworth bandpass filter, which utilized a lower cutoff of 20 Hz and an upper cutoff frequency of 500 Hz. Following filtering, an algorithm was implemented to detect the presence of sEMR by assessing the peak absolute value of the filtered EMG amplitude within the 7 to 50 ms window post-stimulation. Namely, a detected response was considered valid if the peak amplitude exceeded three times the baseline pre-stimulus value (calculated from −50 ms to −7 ms) and surpassed a threshold of 60 microvolts. For participant SCI03, this threshold was set at 30 microvolts because despite exhibiting low-amplitude responses, the participant showed detectable muscle responses based on sEMR shape and latency. To ensure the reliability of the algorithm for detecting sEMR, accuracy was validated through visual confirmation. The cumulative incidence of sEMRs were calculated based on Equation (1).

(1) $$Cumulative\;incidence=\frac{Number\;of\;Occurences\;of\;sEMR\;in\;post-stimulation\;period}{Total\;number\;of\;trials\;until\;reaching\;maximum\;tolerance\;amplitude}*100$$

#### 2.5.2. Recruitment Timing of Muscles Across Different Muscle Groups (Proximal vs. Distal) and Montages

To analyze the recruitment timing of muscles across different montages and between different muscle locations (proximal vs. distal), muscles were ranked using a similar published ranking procedure for lower extremity exploration [20]. We used a cumulative ranking system to provide a quantitative measure of muscle ranking scores across different electrode montages. For instance, if two muscles were activated at the same stimulation intensity, they were assigned an equal rank of 1; if a third muscle was recruited at the next higher intensity, it received a rank of 3, maintaining this rank until reaching the highest tolerable stimulation intensity. Next, all ranks were summed and divided by the total number of muscles and multiplied by 100 to report average recruitment per muscle in percent. We performed this analysis for proximal muscles and distal muscles. The proximal muscle group included BB and TB. The distal muscles group included FDS and EDC muscles (we excluded the FDI muscle group from this analysis due to lack of response for most of the participants across different montages).

#### 2.5.3. sEMR Threshold Intensities and Associated Amplitude Values Across Montages

To analyze differences in sEMR threshold intensities between different montages and participants (SCI vs. control), we quantified the first stimulation intensity for each muscle group that resulted in sEMR in that muscle. Moreover, we quantified sEMR amplitude values at these thresholds (tAmpValues) for each muscle group and montage across participants.

#### 2.5.4. Paired-Pulse Paradigm: Analysis of Differences in Response Attenuation Magnitudes Between Patient Groups and Different Montages

For the paired-pulse analysis, we aimed to compare observations between SCI participants and controls in terms of the muscle response, attenuation or absence thereof following the delivery of the second pulse with respect to the first-pulse response. Initially, we analyzed paired-pulse responses using Montage 1 Single-Site at C7-8 level for all participants. For participants SCI02 and Control02 we had sufficient data to examine the paired-pulse responses across all montages, and we used this observational comparison to identify any specific patterns between participant groups. For each analysis, we calculated the average response across all stimulation intensities for the first and second stimulation pulses. The percentage decreased from the mean response of the first pulse stimulation (1st Pulse Response mean) to the second pulse stimulation (2nd Pulse Response mean) was determined using Equation (2).

(2) $$\%decrease=\frac{1st\;Pulse\;Response\;mean-2nd\;Pulse\;Response\;mean}{Stim1mean}*100$$

#### 2.5.5. Paired-Pulse Paradigm: Effects of Amplitude Modulation on Magnitude of Response Attenuation

To analyze the effects of amplitude modulation on magnitude of attenuation, we computed the ratio of the sEMR response to the first stimulation pulse relative to the response to the second stimulation pulse for Montage 1 Single-Site in all participants. We then applied a linear mixed effect (LME) model for each participant and quantified the coefficient of the regression line fitted on the attenuation ratio for each muscle group with increasing the stimulation intensity.

### 2.6. Statistical Procedures

Given the small sample size, we applied non-parametric methods to ensure the robustness of the analysis. To explore the effects of stimulating different spinal levels on spinally evoked muscle responses (sEMR), a non-parametric method based on ranked data using the aligned rank transform (ART) was first applied to adjust for non-normality. Next, a linear mixed effect (LME) model was used across all participants. To assess the effects of each montage on Incidence of sEMR, an LME model was applied to the aligned rank-transformed data for the SCI and Control groups separately, with Participant as a random intercept. *p*-values for each montage comparison were provided in the model output. Post hoc pairwise comparison was conducted on ranked data to reveal differences between the mean values of each montage.

A separate LME model on aligned rank transformed data compared the Incidence of sEMR between the SCI and Control groups, with Group (SCI vs. Control) as a fixed effect. *p*-values for group differences were reported. A likelihood ratio test compared models with and without a random intercept for Participant for all abovementioned analysis to explore the significance of variability across individuals.

Relationship analysis between the various montage configurations and muscle ranks (a proxy for recruitment timing) across proximal and distal muscles was performed using a Kruskal–Wallis test for SCI and Control participants, respectively. To evaluate differences between groups (SCI and Control) in sEMR threshold intensities across muscles and montages, as well as the corresponding muscle response amplitudes (tAmpValue) at these thresholds, we conducted separate Kruskal–Wallis test. Specifically, we used distinct Kruskal–Wallis test to analyze differences in sEMR and tAmpValues across electrode placement montages. Separate models were fitted for the Control and SCI groups. Post hoc pairwise contrasts were performed to compare mean values across different electrode placement montages using Mann–Whitney test. A Kruskal–Wallis test was conducted to examine differences in percent decrease in second response relative to first response across SCI and Control groups for Paired-Pulse paradigm.

## 3. Results

Each of the participants completed a single visit without any adverse events. Of the 3 SCI participants, SCI01 was omitted from comparison across levels because movement artifacts in the data limited analysis to only two levels (C4-5 and C7-8), leaving an incomplete dataset for evaluation. For comparisons of paired-pulse paradigm across different montages, data were available only for SCI02 and Control 02.

### 3.1. Cumulative Incidence of sEMRs Across Levels

**Figure 2:**
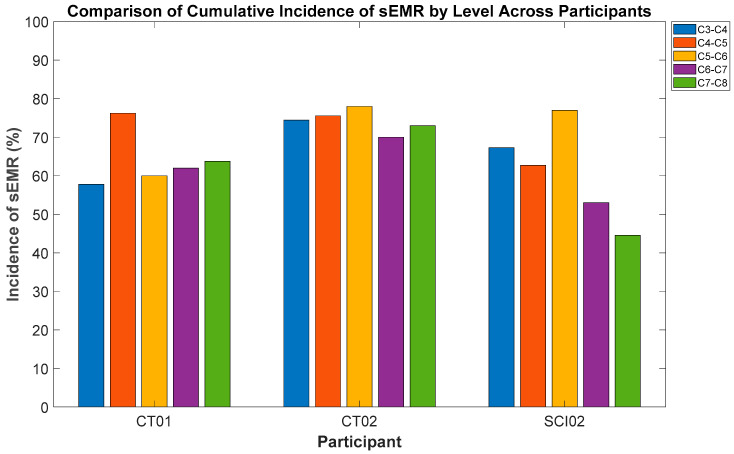
Differences in incidence of sEMR across different spinal levels for participants CT01, CT02 and SCI02. The figure highlights individual variability.

Investigation of the effects of stimulating different spinal levels on cumulative incidence of sEMR showed that the effect was not statistically significant (*n* = 4: 2 participants with SCI and 2 without SCI, F-statistics (1, 12) = 2.37, *p* = 0.14). The results indicated a small negative effect of level on cumulative incidence of sEMR suggesting that as the Level of stimulation moved from rostral levels of spinal column to caudal (from C3-4 to C7-8), the incidence of sEMR decreased, but the effect is minimal as depicted in Figure 2. Moreover, there was a statistically significant effect of Participant (*n* = 4: 2 participants with SCI and 2 without SCI, *p* = 0.04) suggesting that there was variability in the incidence of sEMR between participants. 

### 3.2. Cumulative Incidence of sEMRs Across Montages

For the SCI group (*n* = 3), there was a statistically significant effect of electrode placement montage on the cumulative incidence of sEMR (F-statistics (4, 12) = 4.84, *p* = 0.016). Likelihood ratio test indicated there was not significant variability across participants (χ^2^(1) = 2.02, *p* = 0.15). The cumulative incidence of sEMR was significantly different between all montages with Montage 4 (*p* < 0.05). Also, Montage 1 was trending toward significance compared to Montage 5 (*p* = 0.06). The order of differences between the mean values of sEMR incidence across montages was Montage 1 ~ Montage 3 ~ Montage 2 > Montage 5 > Montage 4.

**Figure 3:**
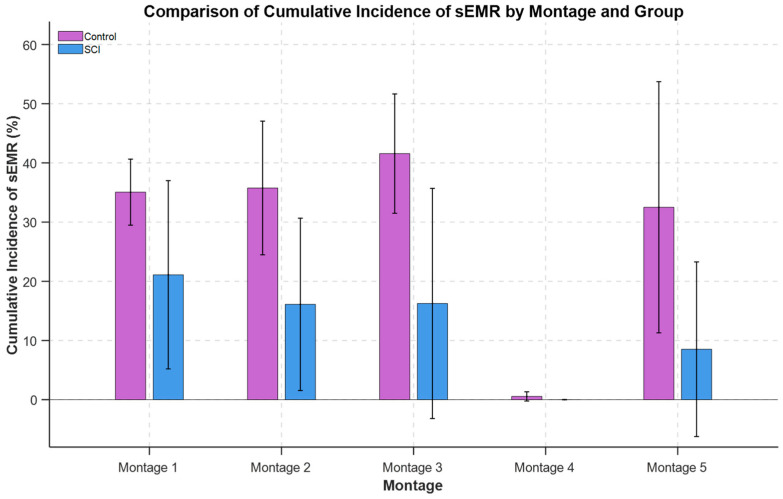
Differences in the incidence of sEMR across montage configurations for participant groups. In the Control group, *Montage 3* showed a noticeably higher total incidence compared to other configurations. In the SCI group, the incidence of sEMR did not differ in a statistically significant manner. The SCI group had a consistently lower incidence of sEMR than the Control group, with on average fewer muscles being recruited across all stimulation intensities.

For the Control group (*n* = 2), there was a statistically significant difference in sEMR incidence between montage configurations (F-statistics (4, 8) = 8.65, *p* = 0.005). The likelihood ratio test indicated there was not significant variability across participants (χ^2^(1) = 1.23, *p* = 0.265). As shown in Figure 3, the cumulative incidence of sEMR was significantly different between all montages with Montage 4 (*p* < 0.001). Also, Montage 3 was trending toward significant compared to Montage 5 (*p* = 0.1). The incidence of sEMR was highest for Montage 3: Montage 3 ~ Montage 1 ~ Montage 2 > Montage 5 > Montage 4.

There was a statistically significant difference in cumulative incidence of sEMR between SCI (*n* = 3) and Control (*n* = 2) groups (F-statistics (1, 24) = 7.76, *p* = 0.010). The likelihood ratio test comparing models with and without a random intercept for Participant showed no significant difference (χ^2^(1) = 0, *p* = 1). The SCI group exhibited a statistically significantly lower cumulative incidence of sEMR compared to the Control group. The cumulative incidence of sEMR averaged across all montages with removal of *Montage 4* as outlier was 15.49% for SCI and 36.22% for Control individuals. The cumulative incidence of sEMR average for each group (SCI and Control) across all montages is depicted in Figure 3.

### 3.3. Recruitment Timing of Muscles Across Different Muscle Groups (Proximal vs. Distal) and Montages

**Figure 4:**
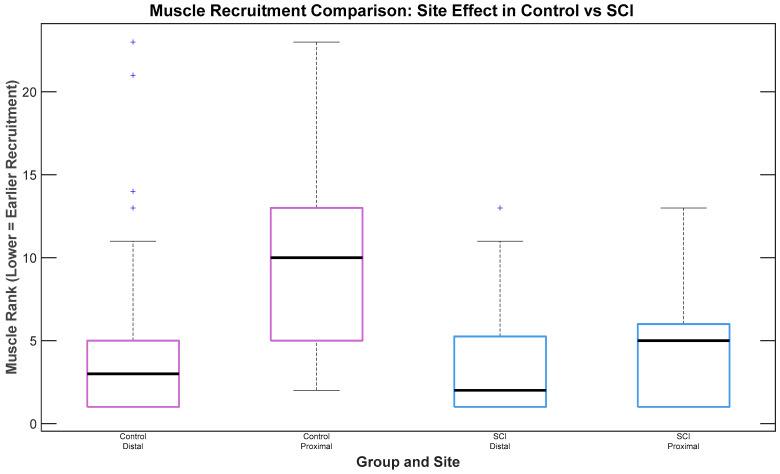
Proximal and distal muscle recruitment timing in participant groups. In the Control group, proximal muscles were recruited later than distal muscles, with a significant difference in recruitment timing. In the SCI group, distal muscles were recruited earlier than proximal muscles; however, there was no statistically significant difference in recruitment timing.

In analysis of the effects of muscle group (proximal vs. distal) and montage on muscle recruitment timing for SCI participants, no statistically significant difference in recruitment was observed between muscle groups (*n* = 3 participants with SCI, *p* = 0.177), although a trend suggested earlier recruitment of distal muscles. For Control participants, a statistically significant difference was observed, with distal muscles exhibiting earlier recruitment than proximal ones (*n* = 2 participants without SCI, χ^2^(1) = 16.82, *p* < 0.001), as depicted in Figure 4. No significant differences in recruitment timing were observed between electrode placement montages.

### 3.4. Differences in sEMR Threshold Intensities Across Montages

**Figure 5:**
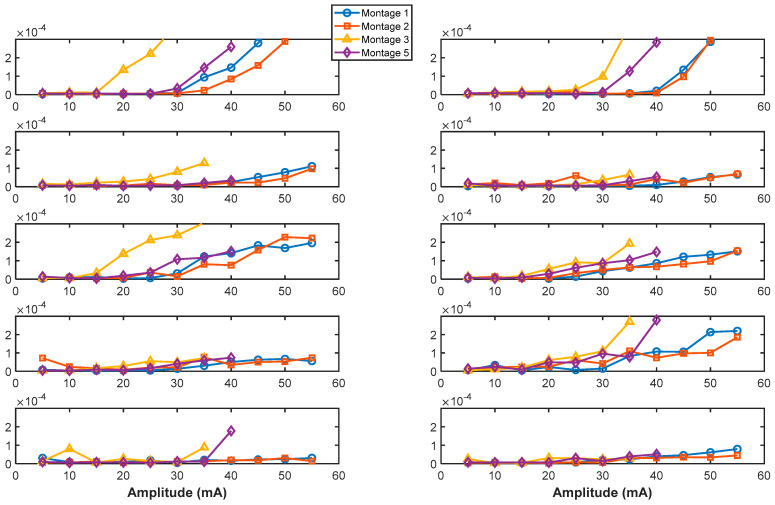
Muscle responses with increasing stimulation intensity for a participant with SCI (SCI02). The montages presented in this figure are Montage 1, Montage 2, Montage 3, and Montage 5. Montage 3 elicited the earliest recruitment threshold for most muscles.

There was a significant difference in sEMR threshold intensities between participant groups (SCI vs. Control), with a (χ^2^(1) = 7.26, *p* = 0.007) showing lower threshold intensity for control participants with respect to SCI participants. For the SCI group, there was no significant difference in sEMR threshold across Montages (χ^2^(3) = 4.33, *p* = 0.23). However, *Montage 3* had the lowest sEMR threshold value among all montages as depicted in Figure 5. For the Control group, there was a statistically significant effect of Montage on sEMR threshold intensities (χ^2^(3) = 9.45, *p* = 0.05). It was shown that *Montage 3* had a statistically significantly lower threshold for stimulation intensity to evoke a spinal motor response across all muscles than *Montage 2*.

### 3.5. sEMR Amplitude Value Across Different Montages

For all participants (*n* = 5: 3 participants with SCI and 2 participants without SCI), there was no statistically significant effect of montage on tAmpValues (χ^2^(4) = 0.04, *p* = 0.9). There was also no significant difference in tAmpValues between participant groups (χ^2^(1) = 0.44, *p* = 0.5).

### 3.6. Paired-Pulse Paradigm: Response Attenuation Across Participant Groups and Different Montages

**Figure 6:**
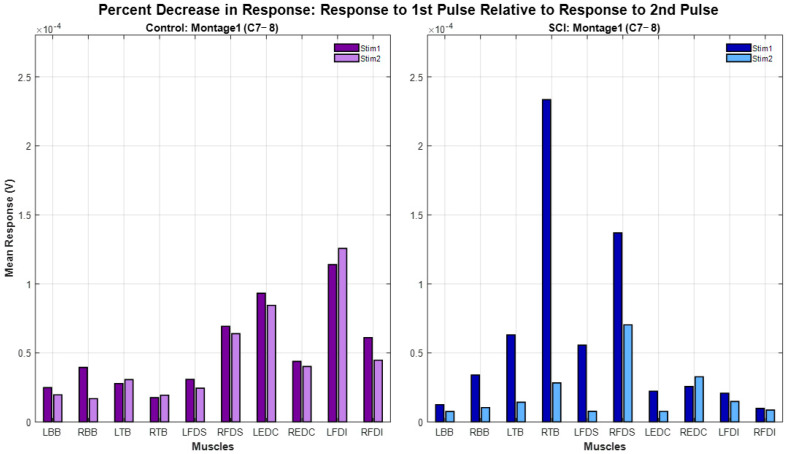
Paired-pulse paradigm results for all participants (SCI and Control) using Montage 1 Single-Site at spinal level C7-8 and an inter-stimulus interval of 50 ms. The sEMR responses were averaged across all stimulation intensities. On average, muscles of SCI participants demonstrated a higher degree of attenuation than those of Control participants across all muscles, with few exceptions (e.g., REDC, RFDI).

The paired-pulse paradigm results of all participants are shown in Figure 6. For the same montage (Montage 1), spinal level (C7-8) and interstimulus interval (50 ms), spinally evoked muscle responses in Control participants exhibited minimal or no attenuation following the delivery of the second pulse (12% ± 20% (mean ± std). Spinally evoked muscle responses in SCI participants exhibited higher attenuation (48% ± 36%). There was a statistically significant difference in percent decrease in the second-pulse evoked response relative to first-pulse response (χ^2^(1) = 4.1143, *p* = 0.0425) between SCI and Control groups.

**Figure 7:**
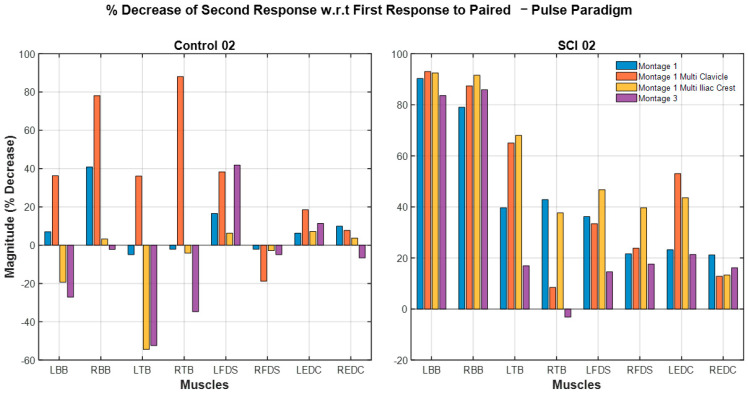
Percent attenuation of sEMR response to the second pulse relative to the first pulse in a paired-pulse paradigm for participants SCI02 and CT02. The SCI participant exhibited higher percentages of attenuation in all muscle groups. For the Control participant, Montage 3 resulted in the greatest attenuation. For the SCI participant, Montage 3 and Montage 2 resulted in higher rates of attenuation than other montages in most muscle groups. For both participants, proximal muscles (BB, TB) generally demonstrated greater percentage attenuation than distal muscle (FDS, EDC, FDI).

The percentage attenuation of the sEMR response to the second pulse relative to the first pulse for all montages in Participants SCI02, and CT02 are depicted in Figure 7. On average, the SCI participant exhibited higher percentages of attenuation (depression) across all montage configurations and all muscles than the Control participant. In the Control participant, *Montage 3* demonstrated the highest attenuation relative to other montages. *Montage 5* showed lower attenuations among all montages for both SCI02 and Control02, and on there appeared to be reduced attenuation in distal muscles (FDS, EDC, FDI group) compared to proximal muscles (BB, TB) for both participants.

### 3.7. Paired-Pulse Paradigm: Analysis of the Effects of Amplitude Modulation on Magnitude of Response Attenuation

Increasing stimulation intensity resulted in a trend of reduced attenuation (ratio of sEMRs to the first pulse/sEMRs to the second pulse) for different muscle groups in the participants. Figure S1 illustrates the muscles for which this attenuation was significant (*p* < 0.05) or weakly significant (0.05 < *p* < 0.15).

**Figure 8:**
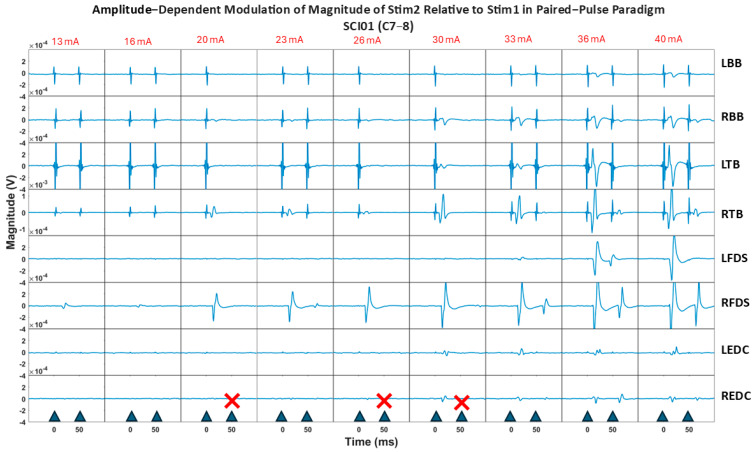
Paired-pulse paradigm sEMRs for SCI01 at increasing stimulation intensities. The relative attenuation between responses to the 1st pulse and 2nd pulse decreased at higher stimulation intensities in the RFDS muscle. The non-physiological stimulation artifact noise diminished for distal muscles, while the physiological response (sEMR) remained consistent. Red cross symbols indicate trials where the second stimulation pulse was not delivered.

Figure 8 illustrates the paired-pulse paradigm responses at increasing stimulation intensities across muscles for SCI01. Results show two key observations emerged as stimulation intensity increased: (1) for certain muscles, such as the RFDS, the relative decrease between responses became gradually smaller, indicating reduced attenuation, and (2) for distal muscles (FDS, EDC groups), the non-physiological stimulation artifact noise (non-electrophysiological muscle response) diminished, while the sEMR (physiological muscle response) remained evident.

## 4. Discussion

The primary objective of this study was to investigate the neurophysiological responses elicited by applying tSCS in different montages and at different spinal levels, and to assess whether these responses differ between individuals with SCI and control participants. Our broader goal was to gain insight and lay the groundwork for personalizing tSCS, ultimately enabling a more targeted and effective therapeutic approach.

### 4.1. Variability in Spinal Level Responsiveness

Our findings revealed participant-specific differences in the responsiveness of muscles to a stimulated spinal level as there was significant variability across subjects. While the analysis results suggested a non-significant trend that stimulating levels rostral in the spinal column provide a higher incidence of sEMRs compared to caudal levels, the limited sample size precludes definitive conclusions, and additional data are required to draw definitive conclusions about potential patterns. Nonetheless, our primary aim was not to establish specific trends but to identify key variables that could guide the personalization of stimulation protocols for future SCI therapy adjustments. These participant-specific differences as well as potential trends in stimulating rostral to caudal levels are crucial information for guiding the placement of epidural implants and targeting the optimal spinal levels to elicit the highest muscle responses, as elaborated in Section 4.6, which could be beneficial if applied in a longitudinal intervention tailored to individual needs. We also highlight that the observed variability across subjects, as well as the existence of a potential rostral to caudal trend, warrants further investigation in future studies with a larger cohort.

### 4.2. Montage-Specific Responses

Of the montages evaluated, *Montage 3* on average demonstrated higher Incidence of sEMR, lower stimulation thresholds needed to elicit the first observable sEMR, and slightly higher amplitudes for sEMRs recruited at the threshold intensity. In computation of the cumulative incidence of sEMR for SCI group, SCI01 was lacking Montage 3, which might have resulted in some bias toward Montage 1. For Control participants, the order from lowest threshold to highest thresholds to elicit the first observable sEMR was Montage 3 ~ Montage 5 < Montage 2 ~ Montage 1 < Montage 4. Moreover, differences in sEMR threshold intensities across montages suggesting intensity-specific responses across montages. Meanwhile, the lack of a significant difference in the amplitude of first sEMR at threshold intensities across montages suggests that the montage configuration does not significantly affect the amplitude of spinally evoked motor responses observed at threshold intensities.

Differences observed between montages in the paired-pulse paradigm provided critical insights into the reflex pathway engagement. Previous studies [22] have shown that high attenuation values (e.g., 53% reduction in second response with respect to the first response, equivalent to an attenuation ratio of 0.047) in the lower extremities of control participants are indicative of posterior root reflexes driven predominantly by afferent fiber activation without significant motor fiber involvement. In this context, the paired-pulse results for our SCI participants suggest greater engagement of afferent nerve fiber reflex pathways than control participants. The activation of α motor neurons by trans-spinal stimulation as suggested by other studies remain a black box. Modeling studies have suggested that upon application of trans-spinal single-pulse stimulation, posterior root fibers of large and small diameters are coactivated along with anterior root fibers, directly activating α motor neurons [23]. It has also been suggested that α motor neurons and interneurons are indirectly activated through trans-synaptic mechanisms and the engagement of spinal circuits. Our results suggest that the recruitment of different fibers or spinal circuits during tSCS is modulated by stimulation intensity [23]. Conversely, lower attenuation observed in control participants may reflect sufficient recovery from post-activation depression or pre-synaptic inhibition at the chosen interstimulus interval (50 ms), enabling unimpeded responses. This highlights the need for further investigation into the frequency-dependent effects of stimulation across different populations, considering variations in neurophysiological recovery dynamics.

### 4.3. Paired-Pulse Paradigm and Stimulation Intensity

The reduced attenuation of responses for specific muscle groups, which differed across participants as the stimulation intensity increased (as shown in Figure S1 and Figure 8), suggests that at maximum tolerable stimulation intensities, for some muscle groups, efferent fibers may be recruited in addition to afferent fibers. This underscores the importance of fine-tuning stimulation intensity for specific therapeutic objectives when targeting specific muscles; excessive stimulation intensity may not always be optimal for the neurophysiological response of interest for a particular muscle group, as it can influence the selectivity of muscle fiber activation.

Moreover, the reduced presence of non-physiological stimulation artifacts (non-physiological responses) as the stimulation moves from proximal to distal muscles aligns with the principle of electrical transmission, wherein signal degradation increases with distance. In contrast, the physiological sEMR responses remained robust, adhering to the all-or-none principle of biological systems consistently observed in both proximal and distal muscles.

Reflex attenuation responses highlighted the variability in reflex pathway engagement across different montage configurations. Additionally, a trend was observed in the degree of attenuation between proximal and distal musculature for SCI02 and CT02; proximal muscles exhibited on average higher levels of attenuation than distal muscles across all montages. This points out potential differences in reflex pathways engagement upon application of stimulation.

### 4.4. Differences Between SCI and Control Participants

The findings that muscle response attenuation differed between SCI and Control participants may suggest variability in neurophysiological recovery periods across the two populations, highlighting the importance of fine-tuning interstimulus intervals in paired-pulse paradigms. Moreover, it emphasizes the valuable information that applying different interstimulus intervals can provide across subject population (SCI, Control), as well as different levels of injury in SCI population. The differences can further elucidate the neurophysiological impact of spinal cord injury and suggest that after injury a new central nervous system is built in which the underlying neurophysiological mechanisms of motor control differ from the (former) intact one in some respects.

### 4.5. Clinical Implications as an Objective Metric

tSCS is both a therapeutic tool and a means to potentially advance our understanding of spine neurophysiology. Identifying a spinal level with greater response could guide the placement of a permanent implant for SCS. The paired-pulse paradigm and other neurophysiological metrics may have the potential to serve as diagnostic tools or biomarkers for tracking neurophysiological changes in rehabilitation. For instance, Figure S2 illustrates differences in response latencies between participants SCI02 and CT02, with consistent delays observed in the LTB muscle for both montages. Such insights can inform rehabilitation strategies by identifying specific neurophysiological deficits and monitoring the effects of interventions over time.

Additionally, our findings underscore the importance of distinguishing between afferent and efferent fiber recruitment in therapeutic contexts. Selectively targeting afferent fibers needs fine-tuning of stimulation intensity. Further clinical studies are needed to evaluate the therapeutic outcomes of activating afferent versus efferent fibers, or a combination thereof, in specific patient populations.

### 4.6. Limitations

This study has several limitations. The small sample size reduces the generalizability of the findings and may limit statistical power. To alleviate this limitation, we employed a statistical approach designed to handle small sample sizes and non-normally distributed data by using non-parametric methods. In the case that we wanted to utilize the linear mixed effect model to capture whether we have variability across participants, we used the aligned ranked transform to handle non-normality. Meanwhile, we acknowledge the small sample size may have a potential impact on the validity of the findings. Yet, we attempted to emphasize our findings in the context of exploration of the impactful variables to enhance personalized treatment. Additionally, the heterogeneity in AIS classification and spinal cord injury levels introduces variability that could influence the results. Furthermore, the inclusion of a limited number of participants of different biological sexes may impact the interpretation of sex-related differences. Future studies with larger, more homogeneous samples are needed to validate these findings.

## 5. Conclusions

Implementing tSCS in a personalized manner would maximize its therapeutic potential. In this study, we showed how different electrode montages and spinal levels can be tailored for optimal stimulation of muscles, and could differ between individuals and participant groups (SCI and Control). We also showed that engagement of dorsal root fibers is sensitive to electrode placement montage.

Personalization of the tSCS technique is important to deliver interventions that are tailored to the physiological responses of individuals. For this approach to succeed, all factors highlighted in this study should be considered, including spinal levels, montages, stimulation intensity, and population-specific and individual-specific differences. However, these findings need to be explored as part of a larger cohort study before making conclusions regarding the reported trends. Furthermore, the neurophysiological effects of other parameters of stimulation such as frequency, waveform type, and pulse-width need to be explored to provide a more comprehensive framework of personalization. Finally, we explored these parameters while participants were at rest; experimental designs are needed to shed light on how stimulation parameters affect neurophysiological responses of muscles while the participants perform tasks.

While the small sample size of our study limits the generalizability of our findings, this work has established a framework for our future research. We plan to develop a data-driven decision-making procedure for tSCS personalization and ultimately evaluate its efficacy using our innovative technology (RISES System) to apply personalized tSCS in larger and more diverse cohorts of participants with various injury profiles. By adopting this patient-centric approach, we will meaningfully evaluate the efficacy of this technique.

## Figures and Tables

**Table 1:** Inclusion and exclusion criteria for SCI participants.

Inclusion criteria Age 18 years or older. Has a non-progressive or traumatic spinal cord injury. American Spinal Injury Association (ASIA) Impairment Scale (AIS) classification A, B, C, or D. Can participate in physical and occupational therapy rehabilitation programs. Is at minimum 12 months post-injury. Can provide informed consent, as evidenced by the teach-back method. Has adequate care partner support to facilitate participation in study. Exclusion criteria Has uncontrolled cardiopulmonary disease or cardiac symptoms (as determined by investigators). Has any unstable or significant medical condition that is likely to interfere with study procedures or likely to confound study endpoint evaluations such as uncontrolled cardiopulmonary disease or cardiac symptoms, unmanaged neuropathic pain, unmanaged autonomic dysreflexia, unmanaged orthostatic hypotension, uncontrolled spasticity. Requires ventilator support. Has an autoimmune etiology of spinal cord dysfunction/injury. Has pressure injury in area(s) that will come into contact with electrodes or that has potential to worsen with study participation. Has an implanted medical device (e.g., cochlear implant, pacemaker, neurostimulator or a powered medication infusion devices such as baclofen pump). Is pregnant, planning to become pregnant or currently breastfeeding. Has concurrent participation in another drug or device trial that may interfere with this study. Has other traumatic injuries such as peripheral nerve injuries, severe musculoskeletal injuries that prevent evaluation of response to or participation in rehabilitation.

**Table 2:** Demographic information of SCI participants.

Subject ID	Age (Yrs)	Sex	Years Since Injury (Years)	Cause of Injury	AIS and NLI at Study Baseline
SCI01	57	Male	2	Windsurfing accident	T12 D
SCI02	64	Male	2	Traumatic fall	C6 B
SCI03	50	Female	11	Sports	C3 A

AIS = American Spinal Injury Association (ASIA) Impairment Scale. NLI = Neurological Level of Injury.

**Table 3:** Montages and electrode characteristics in this Study.

Montage	Cathode Number/Shape/Size	Anode Number/Shape/Size	Anode Location	Cathode Location
Montage 1	1 Circular, 1.25″ Diameter	2 Rectangular (3″ × 4″)	Bilateral Iliac Crest	Single-Site
Montage 2	2 Circular, 1.25″ Diameter	2 Rectangular (3″ × 4″)	Bilateral Iliac Crest	Multi-Site
Montage 3	2 Circular, 1.25″ Diameter	2 Rectangular (3″ × 4″)	Bilateral Clavicles	Multi-Site
Montage 4A	1 Circular (1.25″ Diameter)	1 Rectangular (2″ × 3.5″)	Spinal Level of Interest	Single-Site
Montage 4B	1 Square (2″ × 2″)	1 Square (2″ × 2″)	Spinal Level of Interest	Multi-Site
Montage 5	2 Circular, 1.25″ Diameter	2 Rectangular (3″ × 4″)	Bilateral Iliac Crest	Single-Site

## Data Availability

Data are available upon request.

## Supplementary Materials

The following supporting information can be downloaded at: https://www.mdpi.com/article/10.3390/bioengineering12040410/s1, Figure S1: Effect of Amplitude on Ratio of Post Stim1 to Post Stim2 Response (Only significant or weak significant Negative Trends); Figure S2: Comparison of Response Latencies Between CT02 and SCI02.
