# Supplementary material for: Optimizing Transcutaneous Spinal Cord Stimulation: An Exploratory Study on the Role of Electrode Montages and Stimulation Intensity on Reflex Pathway Modulation
Source: Bioengineering (Basel). 2025 Apr 12;12(4):410. doi: 10.3390/bioengineering12040410 (PMC12024636; doi:10.3390/bioengineering12040410)
Supplement: Supplementary file 1 [file bioengineering-12-00410-s001.zip › bioengineering-3536756-supplementary.pdf]

Supplementary Material:

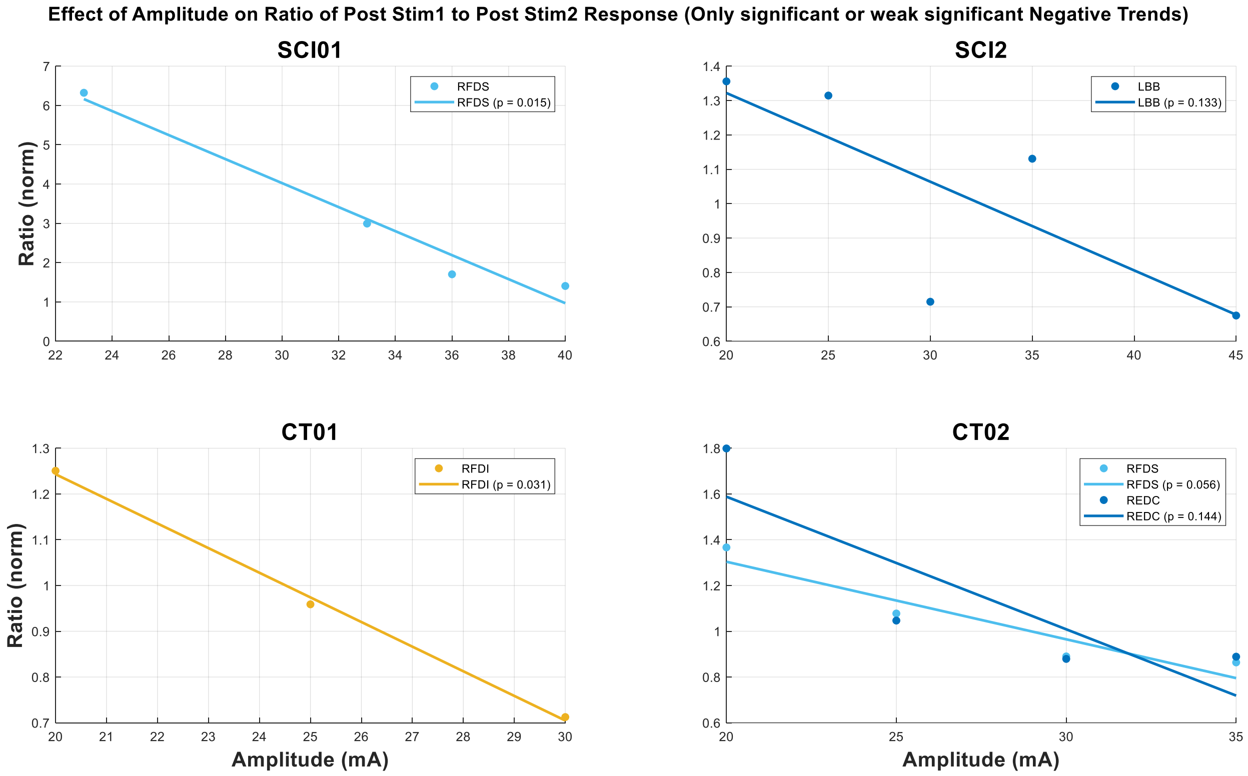

Supplementary Figure S1. Muscles in which increasing amplitude had significant (p <0.05) or weak significant (0.05<p <0.15) effects on attenuation (ratio of sEMR response to first pulse/sEMR response to second pulse).

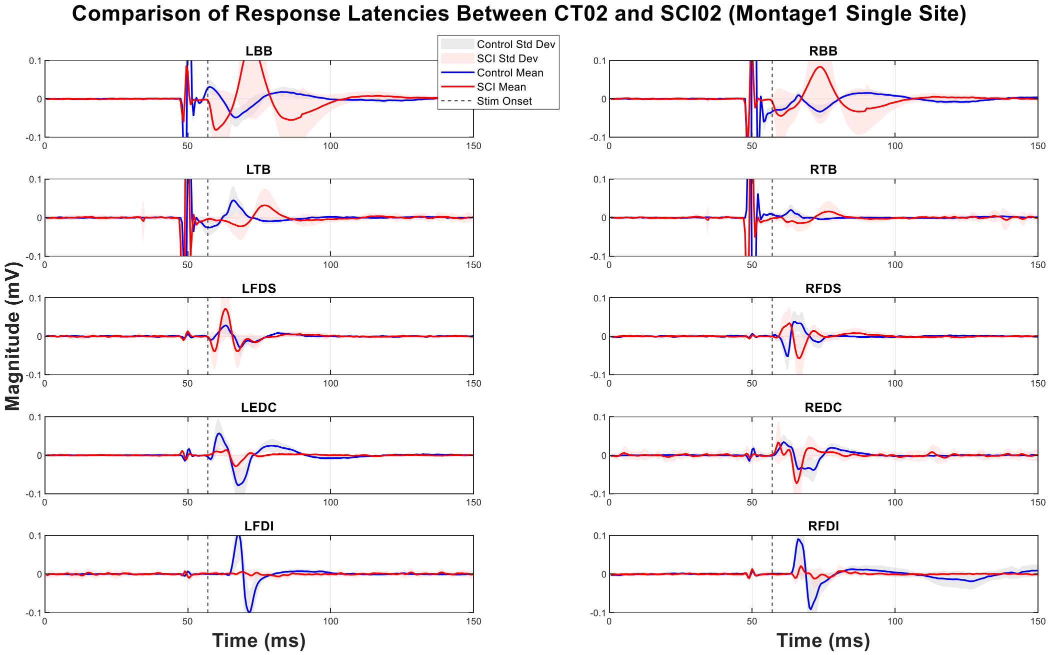

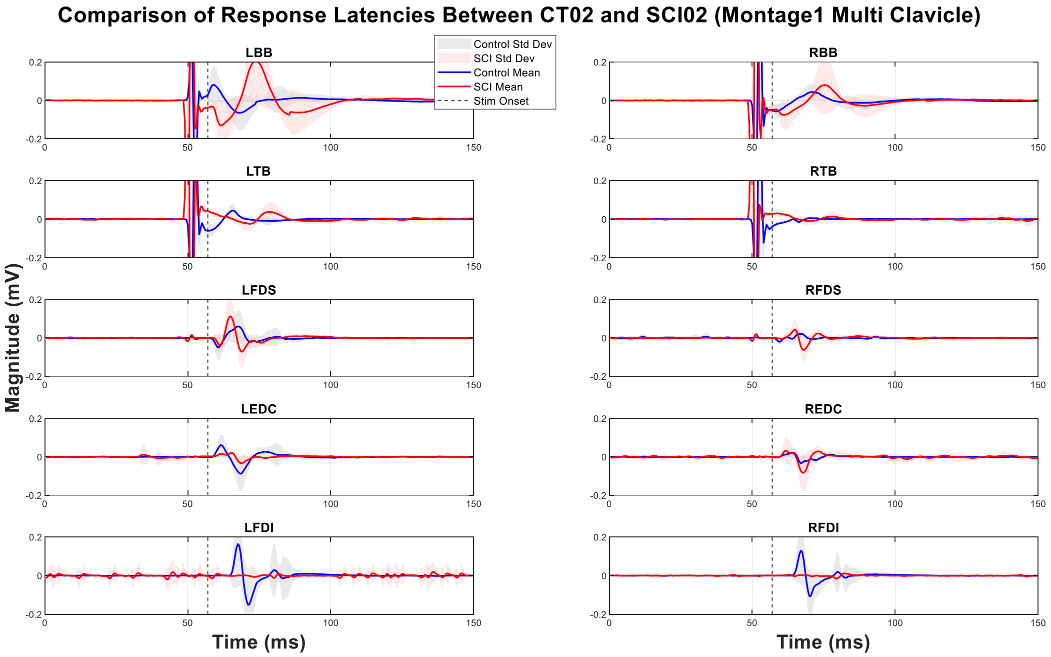

Supplementary Figure S2. Response latencies of proximal and distal muscles in participants CT02 and SCI02 subjects with standard deviation shades across all stimulation intensities. Notably, the proximal muscle on the right side of SCI subject shows a delay in response. Methods such as single-pulse tSCS can give of insights on these individualized differences and may be useful in the future for tracking the effects of various interventions and for
